# Characterization of a branched lipopeptide candidate vaccine against influenza A/Puerto Rico 8/34 which is recognized by human B and T-cell immune responses

**DOI:** 10.1186/1743-422X-8-309

**Published:** 2011-06-16

**Authors:** Liz Samayoa, Francisco Diaz-Mitoma, Ali Azizi

**Affiliations:** 1 Infectious Disease and Vaccine Research Center, Children's Hospital of Eastern Ontario Research Institute, 401 Smyth Rd, Ottawa, ON, K1H 8L1, Canada; 2 Department of Pathology and Laboratory Medicine, University of Ottawa, 451 Smyth Rd, Ottawa, ON, K1H 8M5, Canada; 3 Department of Microbiology and Immunology, University of Ottawa, 451 Smyth Rd, Ottawa, Ontario, K1H 8M5, Canada; 4 Sudbury Regional Hospital Foundation, 41 Ramsey Lake Road, Sudbury, ON, P3E 5J1, Canada

## Abstract

The use of synthetic peptides as immunogens represents an exciting alternative to traditional vaccines. However, to date most of these synthetic peptides are not highly immunogenic. The lack of immunogenicity might be addressed by conjugation between T or B cell epitopes with universal or immunodominant T-helper epitopes. The construction of lipidated peptides, branched peptides, or designs combining both of these elements might enhance the immunogenicity, as they might target Toll-Like Receptors and/or mimic the 3-dimensional structure of epitopes within the native protein. Herein, a recognized peptide immunogen based on the hemagglutinin protein of A/Puerto Rico/8/34 was chosen as a backbone and modified to evaluate if the construction of branched peptides, lipidation, the addition of cysteine residues, or mutations could indeed alter epitope reactivity. Screening the different designs with various antibody binding and cellular assays revealed that combining a branched design with the addition of lipid moieties greatly enhanced the immunoreactivity.

## Background

Influenza virus is a leading cause of disease worldwide, affecting up to 500 million people each year. Most commonly spread by aerosols, symptoms of viral infection are varied and can include headaches, sneezing, fever, and general discomfort. Although most people are able to clear the virus without any major complications, infections can progress to pneumonia in children, the elderly and otherwise immunocompromised patients, causing an estimated 500,000 deaths per season. These statistics change drastically during pandemics, as was seen during the recent H1N1 outbreak wherein a single strain of influenza caused an estimated 18 500 deaths [1]. There are vaccines available to prevent influenza infection, and it is recommended that all patients above the age of 6 months receive a yearly vaccination [2]. However, the vaccines currently on the market suffer from several weaknesses. The viral surface glycoproteins (hemagglutinin and neuraminidase) regularly undergo amino acid changes that often lead to new variant strains [3]. As a result, the seasonal influenza vaccine must be reformulated and readministered on a yearly basis. Production of vaccine is in itself a laborious and costly process; each strain included in the formulation needs to be harvested from the allantoic fluid of embryonated chicken eggs, purified and inactivated. Production can take up to 9 months, and vaccines need to be stored at temperatures below 8 degrees Celsius, and cannot be given to individuals with egg allergies [4].

An interesting alternative to "traditional" vaccines would be the use of synthetic viral peptides as immunogens. In terms of manufacturing, these compounds can be rapidly and relatively affordably mass-produced. Additionally, the need for refrigeration is eliminated as peptides can be stably kept as dry powder for long periods of time. From an immunological point of view, vaccination with short and well-defined peptides may be preferential to immunizing with whole viral proteins. Since the ability to induce humoral and cellular immune responses is limited to specific regions (epitopes) within any given protein, restricting vaccine components to immulogically important epitopes could result in more focused and thus stronger cellular and humoral responses [3,5]. On the other hand, peptide immunogens may suffer from inherent weaknesses. Proteases may degrade the peptides before they reach their intended targets, and there is also the risk of formation of dimers and other types of aggregates (via reactive terminal cysteine residues) [6]. Of greatest significance is the fact that short peptides elicit only moderate immune responses at best [5].

However, there are several approaches that might increase the stability and immunogenicity of peptide immunogens. Coupling peptides to lipid moieties has been found to increase the biological half-life of synthetic peptides [7]. The addition of lipid moieties has also been shown to have an adjuvant-like effect, enhancing otherwise moderate immune responses; lipidated influenza peptides were found to enhance specific CD8^+ ^immune responses [8]. Furthermore, addition of lipid chains to peptides resulted in more efficient cytosolic uptake and prolonged presentation events [9], and lipidated peptides were found to be better immunogens than non-lipidated equivalents in terms of inducing HCV-specific humoral immune responses in HCV-naïve blood donors [10,11]. As opposed to chemical adjuvants such as alum, lipid moieties have been tested in human trials with few or no side effects [12]. Although the precise mechanisms whereby lipid side chains achieve their adjuvanticity have to date not been elucidated, various studies suggest that Toll-like receptor 2 (TLR-2) is involved in binding lipidated peptides [13,14]. Of particular importance to a potential influenza vaccine candidate, this receptor is expressed on the epithelia of air passages. The lipid-TLR-2 interaction has been found to lead to activation of dendritic cells as evidenced by the up-regulation of MHC class II molecules and to induce nuclear factor kappa-light-chain-enhancer of activated B cells (NF-κB)[6,15], as well as trigger inflammatory signalling pathways in macrophages resulting in the production of tumor necrosis factor-alpha (TNF-α), interleukin-6 (IL-6), and monocyte chemotactic protein-1 (MCP-1) [16].

Linking several epitopes in a dendrimer-like arrangement may represent another method of enhancing the stability of peptide immunogens; that is, constructing poly-peptide structures or Multiple Antigen Peptides (MAPs). Increased molecular size has been correlated to decreased degradation and thus a longer serum half-life [17]. Both computer modeling and electrophoretic analysis also suggest that linking the influenza T-helper and B-cell epitopes used herein orients the peptides in a more compact and globular shape than when these are not linked [7]. Moreover, linked epitopes in a branched MAP construct should attain a more natural conformation, which may in turn lead to enhanced recognition and increased binding with B-cells. Therefore, this strategy is another technique to make potential epitope-based vaccines more immunogenic; joining T-helper epitopes to B-cell epitopes leads to stronger immune responses, possibly as mediated by T-B-cell cooperation [5,7]. Indeed, previous results suggest that the manner in which peptides are oriented have an important effect on their immunogenicity; enhanced reactivity is achieved when epitopes are arranged such that native antigenic conformation is mimicked [18,19]. Furthermore, having multiple antigens per molecule means that more epitopes are available to interact with antigen presenting cells (APCs), and may thus activate stronger immune responses [20].

Brumeanu's group immunized mice with a short synthetic influenza peptide consisting of a T-helper epitope linked to a synthetic and immunodominant influenza B-cell epitope, based on the H1N1 strain A/Puerto Rico/8/34. Although only modest humoral and cellular immune responses were detected in vaccinated animals, no significant responses were detected in the group that had been administered equimolar amounts of non-linked T and B peptides [7]. With this in mind, we attempted to modify the simple linked T-B peptide design such as to build a more effective immunogen. In the present study, we compare the effect that lipidation, the construction of MAPs, the addition of a cysteine residue, and certain mutations have on immunoreactivity. Using a bioinformatic approach, we designed and synthesized an array of 16 unique peptides, representing the two previously studied antigenic sites from the hemagglutinin protein of the influenza strain A/Puerto Rico/8/34. Various *in vitro *assays were used to screen individual constructs for differences in reactivity in both humoral and cellular assays, for future use in animal studies. Commercial strain-specific anti-influenza sera, and immune human plasma and peripheral blood mononuclear cells (PBMCs) were used to characterize differences in the reactivity of peptides. Our *in vitro *humoral and cellular results suggest that the most critical factor to be considered in terms of improving immunoreactivity is mimicking a more native epitope conformation. This can be easily achieved while still maintaining a relatively simple design by constructing branched and lipidated peptides.

## Materials and methods

### Peptide synthesis and purification

Peptides based on the influenza A/Puerto Rico/8/34 H1N1 strain hemagglutinin (HA) were synthesized by the solid phase method on a Symphony Peptide Synthesizer (Protein Technologies, Tucson AZ). Briefly, amino acids were coupled in sequential format from the COOH terminus using standard N-(9 fluorenyl)methoxycarbonyl (FMOC) chemistry. Peptide stock solutions were then prepared by dissolving lyophilized preparations in double distilled water at a concentration of 5 mg/ml and stored at -80°C until use.

Altogether 16 peptides were constructed; a simple construct consisting of a T-helper epitope (^110^HA^120^, sequence SFERFEIFPKE) linked by two glycine spacers (GG) to a B-cell epitope (^150^HA^159^, sequence WLTEKEGSYP), and 15 modifications thereof. Peptides that were lipidated had palmitic acid (CH_3_(CH_2_)_14_COOH) added at the N-terminus; multiple antigen peptides (MAPs) were constructed by creating branching points with lysine residues; mutated peptides were designed by aligning all post-1934 H1N1 sequences available from the Los Alamos National Laboratory Influenza Sequence Database (LANL ISD) [21], and comparing them using a proprietary alignment program. Protein analysis was done using the National Center for Biotechnology Information Basic Local Alignment Search Tool (NCBI BLAST) [22].

A non-influenza peptide (a T-cell epitope from the HIV gag protein, with sequence HKGRPGNFLQNRPEPTAP) was obtained from the National Institutes of Health AIDS Research and Reference Reagent Program (Rockville, Maryland) and was included in all assays at the same concentration as the influenza peptides as a negative control. Likewise, recombinant influenza hemagglutinin protein from the A/New Caledonia/20/99 strain (Protein Sciences, Meriden, Connecticut) was included in each assay as a positive control.

### Human samples

Approximately 100 ml of peripheral blood was obtained via venipuncture from 16 healthy donors, in accordance with the guidelines set forth by the Children's Hospital of Eastern Ontario (CHEO, Ottawa, Ontario). Consent forms were read and signed by each volunteer. For confidentiality purposes, donors were assigned a number between 1144 and 1161 (numbers 1152 and 1153 were assigned, but donors failed to show for the blood draw hence these numbers were skipped). The group of volunteers consisted of 8 females and 8 males ranging in age from 23 to 55 years old, who had been immunized with the 2007/2008 FluViral influenza vaccine (GlaxoSmithKline, London, UK). Blood was collected in 8 ml Cell Preparation Tubes (CPTs) with sodium citrate as an anticoagulant (BD, Franklin Lakes, New Jersey). The CPTs were allowed to sit at room temperature for 30 minutes and were gently mixed by inversion prior to density gradient centrifugation at 1,700 Relative Centrifugal Force (RCF) for 25 minutes without brake. The top layer of plasma was removed from the CPTs by pipetting and placed at -80°C. The lower layer of peripheral blood mononuclear cells (PBMCs) was then isolated by washing twice with 10 ml Phosphate Buffered Saline (PBS) (Thermo Fisher Scientific, Waltham, Massachusetts). PBMCs were counted in Türk stain (0.01% gentian violet, 1.0% acetic acid) and aliquoted in freezing media consisting of 90% heat-inactivated fetal bovine serum (FBS) and 10% dimethyl sulfoxide (DMSO) (Sigma-Aldrich, St. Louis, Missouri) at cell concentrations ranging between 3 × 10^6 ^to 10 × 10^6 ^PBMCs/ml prior to storing at -80°C.

### Viruses & cells

Influenza A/New Caledonia/20/99 (H1N1) and A/Puerto Rico/8/34 (H1N1) viruses were acquired from CHEO and the American Type Culture Collection (ATTC, Manassas, Virginia), respectively. Viruses were propagated via inoculation into the allantoic cavity of 10-day-old embryonated chicken eggs (Canadian Food Inspection Agency, Ottawa, Canada). Virus stocks were stored at -80°C until use.

Madin-Darby Canine Kidney (MDCK) cells (ATTC, Manassas, Virginia) were grown in Iscove's Modified Dulbecco's Medium (IMDM) (Thermo Fisher Scientific, Waltham, Massachusetts) supplemented with 10% (v/v) FBS and 1% penicillin/streptomycin (Cellgro, Manassas, Virginia) in a humidified 5% CO_2 _atmosphere at 37°C.

### ELISA

Screening by ELISA was performed as previously described [23,24]. Briefly, EIA/RIA Stripwell 96-well plates (Corning Incorporated, Corning, New York) were coated with 100 μl per well of recombinant A/New Caledonia/20/99 hemagglutinin protein (Protein Sciences, Meriden, Connecticut) diluted to 1 μg/ml in PBS or individual influenza or HIV gag diluted to 10 μg/ml in PBS, sealed with adhesive film and incubated overnight at 4°C.

Plates were washed 6 times with 300 μl/well of PBS/0.05% Tween20 (Sigma-Aldrich, St. Louis, Missouri) and incubated for 1 hour at 37°C after the addition of 300 μl per well of blocking buffer (either PBS/5% FBS for plates used for influenza strain-specific sheep sera, or PBS/10% FBS/2% skim milk for plates used for human plasma). The plates were washed as above and incubated 1.5 hours at 37°C after the addition of 100 μl per well of influenza strain-specific antiserum (sheep serum positive for neutralizing antibodies against A/New Caledonia/20/99, obtained from the National Institute for Biological Standards and Controls, Herts, UK) diluted to 1/100 in PBS/5% FBS, or heat-inactivated human plasma diluted to 1/100 in PBS/10% FBS/2% skim milk. After this incubation, the plates were washed again as previously and incubated 1 hour at 37°C with 100 μl per well of 1/10,000 rabbit anti-sheep IgG HRP-conjugate (Abcam, Cambridge, Massachusetts) or 1/5000 goat anti-human IgG HRP-conjugate (Abcam, Cambridge, Massachusetts). Plates were washed again and incubated at room temperature with 100 μl per well TMB One Component HRP Microwell Substrate (SurModics, Eden Prairie, Minnesota). The colorimetric reaction was stopped after 8 minutes by adding 100 μl per well of 450 nm Stop Reagent for TMB Microwell Substrates (SurModics, Eden Prairie, Minnesota). The optical density of each well was then read at 450 nm on a 3550-UV microplate reader (Bio-Rad Laboratories, Hercules, California).

### Competitive microneutralization assay

The competitive microneutralization assay was a modified version of a protocol previously described by our group [25]. Briefly, heat-inactivated human plasma samples were diluted to a concentration of 1/80 in 2% FBS/IMDM. 50 μl of plasma were combined with 50 μl of individual influenza or HIV gag peptide at 50 μg/ml or 50 μl of recombinant A/New Caledonia/20/99 hemagglutinin protein at 3 μg/ml, and incubated 1 hour at 37°C. 50 μl of plasma/peptide mixture were then added to 50 μl of influenza virus (15xTCID_50 _of A/Puerto Rico/8/34 virus in 2% FBS/IMDM) in a flat-bottom 96-well plate (Corning Incorporated, Corning, New York). The following controls were in place on every plate: virus control (50 μl 2% FBS/IMDM plus 50 μl virus); cell control (100 μl 2% FBS/IMDM); no peptide control (25 μl diluted plasma plus 25 μl 2% FBS/IMDM plus 50 μl virus). After these 96-well plates were incubated for 1.5 hours at 37°C, 1 × 10^5 ^freshly trypsinized MDCK cells were added to each well. Plates were incubated overnight for 18-22 hours in a humidified 5% CO_2 _atmosphere at 37°C.

The following day, media was removed by inversion and plates were washed once with 200 μl of PBS. Cells were then fixed by the addition of 100 μl of cold 80% acetone to each well for 10 minutes. After removal of acetone by inversion, the plates were air-dried for 20 minutes. Plates were then washed 5 times with 300 μl PBS/0.05% Tween20 and incubated for 1 hour at room temperature with 100 μl per well of biotinylated influenza A anti-NP antibody (Chemicon International, Temecula, California) at a dilution of 1/2000 in 5% FBS/PBS. Plates were washed as before and incubated for 1 hour at room temperature after the addition of 100 μl per well HRP-conjugated streptavidin (Upstate, Temecula, California) at a dilution of 1/10,000 in 5% FBS/PBS. Plates were once again washed and incubated at room temperature with 100 μl per well TMB One Component HRP Microwell Substrate. The colorimetric reaction was stopped after 12 minutes by adding 100 μl per well of 450 nm Stop Reagent for TMB Microwell Substrates. The optical density of each well was read at 450 nm using an Emax microplate reader (Molecular Devices, Sunnyvale, California).

### Competitive plaque reduction assay

A previously described and tested protocol was modified by our group [26]. Briefly, freshly trypsinized MDCK cells were washed and resuspended in supplemented IMDM at a concentration of 1.125 × 10^5 ^cells/ml. Three millilitres of cell suspension were then seeded into each well of 6-well tissue-culture treated polystyrene, flat-bottom plates (BD, Franklin Lakes, New Jersey). After 48 hours incubation in a humidified 5% CO_2 _atmosphere at 37°C, cells were verified to have reached 90-95% confluency.

Heat-inactivated A/New Caledonia/20/99-specific sheep antiserum or human plasma samples were diluted to a concentration of 1/10 or 1/80, respectively in supplemented IMDM. Individual influenza or HIV peptides were diluted to 50 μg/ml while recombinant A/New Caledonia/20/99 hemagglutinin protein was diluted to 10 μg/ml. 50 μl diluted peptide or diluted protein were added to 50 μl diluted sheep serum or diluted human plasma and incubated for 1 hour at 37°C. 100 μl of influenza virus diluted to 120 plaque-forming units (PFUs) were then added to the mixture from the previous step, and once again incubated for 1 hour at 37°C.

Pre-seeded 6-well plates were washed twice with warm PBS, and 100 μl of mixture were added to the appropriate well. Upon addition to the sample/peptide mixture the influenza virus was diluted by a factor of 2, therefore 60 PFUs were added to each well. To verify that this was indeed the amount of virus added, a virus control (100 μl of virus diluted to 60 PFUs) was run for each sample. A cell control (100 μl PBS alone), and a no peptide control (wherein 50 μl PBS were added to 50 μl serum/plasma prior to the first incubation) were also included for each serum/plasma tested. Plates were incubated for 1 hour at 37°C in a humidified 5% CO_2 _atmosphere, and were gently rocked back and forth every 15 minutes throughout the incubation period to ensure even distribution of virus.

At the end of the incubation, 3 ml of warm overlay were gently added to each well. Overlay consisted of equal parts supplemented 2x Minimum Essential Medium (VWR, West Chester, Pennsylvania) (2x MEM containing 2% penicillin/streptomycin, 2% L-Glutamine (Invitrogen, Carlsbad, California) and 0.25% sodium bicarbonate (Sigma-Aldrich, St. Louis, Missouri)) and 1.3% agarose (Invitrogen, Carlsbad, California) dissolved and melted in 100 ml of distilled water. Once MEM/agarose solution had cooled to 37°C, 0.9 μg/ml of TPCK-trypsin was added.

After 20 minutes at room temperature, the overlay had solidified and plates were returned to the incubator for 3 days. Carnoy's fixative (three parts methanol to one part acetic acid) was then added to each well for 30 minutes, and discarded by inversion. The agarose discs were removed by carefully rinsing plates under a gentle stream of tap water, and a 0.1% crystal violet solution was used to stain the wells. After 30 minutes, stain was rinsed off, plates were allowed to air dry, and plaques were counted.

### Cellular proliferation assay

A protocol previously used in our laboratory was optimized for use with human cells [23,24,27,28]. Briefly, frozen PBMCs were thawed in a 37°C water bath and washed twice with 10 ml warm Roswell Park Memorial Institute (RPMI) medium (Thermo Fisher Scientific, Waltham, Massachusetts) supplemented with 10% (v/v) FBS and 1% penicillin/streptomycin. Viable cells were counted by Trypan blue dye exclusion, and cell suspensions were prepared to a concentration of 2.0 × 10^6^cells/ml. 100 μl of these cell suspensions were added to 100 μl of each stimulant (individual influenza or HIV gag peptide at 10 μg/ml or recombinant A/New Caledonia/20/99 hemagglutinin protein at 1 μg/ml). All conditions were tested in triplicate in a round-bottom 96-well plate (BD, Franklin Lakes, New Jersey). Unstimulated cells and cells stimulated with PMA/ionomycin were also included for each donor as negative and positive controls, respectively.

Plates were incubated at 37°C in a humidified 5% CO_2 _atmosphere for 48 or 120 hours (PMA/ionomycin-stimulated cells and peptide/recombinant protein-stimulated cells, respectively). At these time points, 50 μl of [methyl-^3^H] thymidine (Amersham, Amersham, UK) diluted 1/100 (to give 0.01 mCi/ml) in supplemented RPMI were added to each well. Plates were then returned to the incubator for 16-18 hours.

Cells were then harvested onto glass-fibre filter mats (PerkinElmer, Waltham, Massachusetts) using an automated cell harvester (Tomtec, Hamden, Connecticut). The filter mats were allowed to air dry overnight, and were then placed in a plastic sample bag and saturated with Betaplate scint scintillation fluid (PerkinElmer, Waltham, Massachusetts). Radioactivity was counted on a Wallac 1450 Microbeta Plus Liquid Scintillation and Luminescence Counter (PerkinElmer, Waltham, Massachusetts).

### ELISpot assay

IFN-γ ELISpot assay was performed as previously described [23,28]. Briefly, sterile 96-well MultiScreenHTS filter plates (Millipore, Billerica, Massachusetts) were activated by the addition of 15 μl of sterile-filtered 35% ethanol per well. After 1 minute at room temperature the plates were washed 5 times with 200 μl PBS, and 100 μl of Anti human IFN-γ mAb (Mabtech, Nacka Strand, Sweden) at a concentration of 10 μg/ml in PBS were added to each well. Coated plates were incubated overnight at 4°C.

The following day, coating antibody was removed by washing the plates 5 times with 200 μl per well using sterile PBS. Plates were blocked for 1 hour at room temperature with 200 μl per well supplemented RPMI. After the incubation, blocking medium was removed by inversion and the cell suspensions containing the appropriate stimulatory agents were added to each well as follows.

Frozen PBMCs were thawed and counted using the same method as above, and cell suspensions were prepared at a concentration of 2.0 × 10^6^cells/ml. One hundred microliters (2.0 × 10^5^) cells were then added to each well and stimulated with individual influenza peptides, HIV gag peptide, or recombinant A/New Caledonia/20/99 hemagglutinin protein (diluted to a concentration of 10 μg/ml and 4 μg/ml respectively in supplemented RPMI). Unstimulated cells were used as a negative control and PMA/ionomycin stimulated cells were used as a positive control. All conditions were tested in duplicate for each donor. The plates were placed in a plastic container lined with ddH_2_O-moistened paper towel. This container in turn was placed in a humidified incubator with 5% CO_2 _at 37°C and incubated for 48 hours.

Following this incubation, the cells were decanted and the plates were washed 5 times with 200 μl per well of sterile PBS. Biotinylated anti-human IFN-γ (Mabtech, Nacka Strand, Sweden) was diluted to a concentration of 1 μg/ml in PBS/0.5% FBS, and added at 100 μl per well during an incubation of 2 hours at room temperature. The plates were washed as before and 100 μl of streptavidin-ALP (Mabtech, Nacka Strand, Sweden) diluted 1/1000 in PBS/0.5% FCS were added to each well. Plates were incubated for 1 hour at room temperature and washed one more time as before. After 100 μl of BCIP/NBT alkaline phosphatase substrate (Mabtech, Nacka Strand, Sweden) were added to each well, the plates were incubated in the dark at room temperature for 20 minutes. Colour development was stopped by thoroughly washing the plates under running tap water. The plates were allowed to dry, and spots were counted using a dissection microscope at a magnification of 40×.

### Statistical analysis

Results are expressed as mean ± SEM. Statistical significance of the human donor data was evaluated using the Mann-Whitney t-test, where a *p *value equal to or less than 0.05 was considered significant when compared with the HIV gag peptide control.

## Results

### Characterization of peptide immunogens

**Figure 1:**
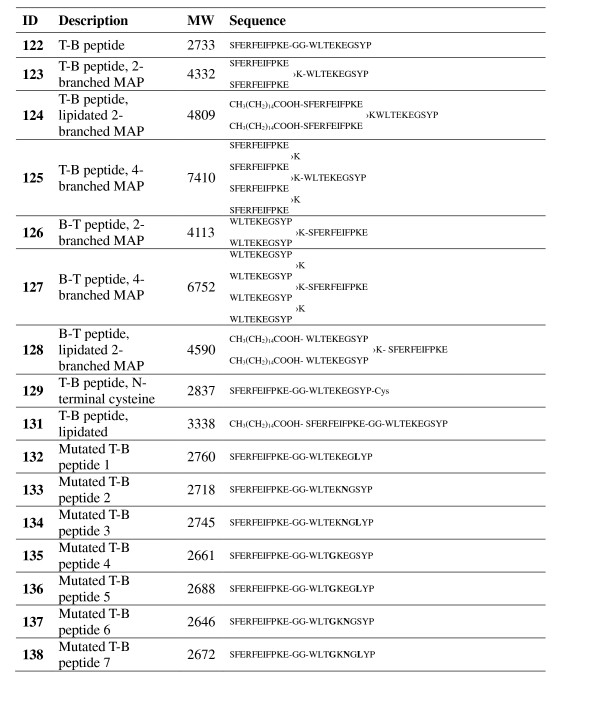
**Sixteen synthetic peptides consisting of a T-helper epitope (SFERFEIFPKE) linked to an immunodominant B-cell epitope (WLTEKEGSYP) based on the hemagglutinin of influenza virus A/Puerto Rico/8/34 were synthesized**. Peptide 122 was a simple, unmodified T-B diepitope construct. Peptides 123-128 are multiple antigen peptides (MAPs, as depicted schematically in Figure 2), where 〉K designates a lysine branching point; peptides 124, 128 and 131 are lipidated with palmitic acid, as indicated by *CH_3_(CH_2_)_14_COOH*; peptides 132-138 are mutated versions of the original A/Puerto Rico/8/34 T-helper and B-cell epitopes, with each mutation shown in bold type (as summarized in Figure 1).

Sixteen synthetic peptides representing the hemagglutinin protein of A/Puerto Rico/8/34 H1N1 strain of influenza were designed. The parent design of these short immunogens consisted of a T-helper epitope linked by two glycine spacers to a B-cell epitope (T-B peptide). Fifteen variations of this basic construct were synthesized; 6 different MAPs, a T-B peptide with an added N-terminal cysteine residue, a lipidated T-B peptide, and 7 mutated versions of the T-B peptide (Figure 1).

**Figure 2:**
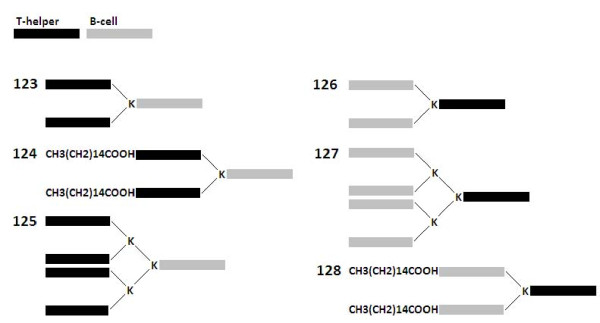
**Schematic depiction of multiple antigen peptides (MAPs)**. Specifically, T-helper and B-cell represent the T-helper epitope SFERFEIFPKE and B-cell epitope WLTEKEGSYP, respectively, both based on the hemagglutinin of influenza virus A/Puerto Rico/8/34. CH3(CH2)14COOH depicts the addition of a palmitic acid lipid moiety, while K represents a lysine residue used as a branching point.

Branching peptides, or MAPs, were generated by incorporating lysine residues into the constructs between the T and B epitopes. Altogether, three different types of MAPs were designed: a construct with one lysine branching point and two chains coming off the backbone, a similar construct that was lipidated with palmitic acid at the N-terminus of each of the two branches, and a larger construct with two lysine branching points and thus four chains coming off the backbone (Figure 2). Each of the three types of MAPs was synthesized using the B-cell epitope as a backbone and the T-helper epitope as branches, and vice versa, for a total of 6 unique designs.

In order to design the mutated peptides, the HA sequences of all H1N1 genomes from after 1934 were downloaded from the LANL ISD [21]. When these sequences were aligned and compared, it was found that the T-helper epitope had been conserved. Conversely, the B-cell epitope had three variable sites: WLT[E→G]K[E→N]G[S→L]YP (where variable sites are shown as [original A/Puerto Rico/8/34 residue→divergent residue]). All 7 possible mutations of the original epitope were synthesized and these were reverse-analysed using the NCBI BLAST program [22] to determine the number of influenza genomes that each unique epitope could be found in (Table 1). Comparing the generated epitopes to the protein sequences available in the NCBI database revealed that not all epitope mutations were generated at the same frequency. In fact, three of the mutated epitopes were very favourable: the T-cell epitopes of constructs 138 (mutated at all three variable positions), 134 (mutated at the second and third variable positions), and 133 (mutated at the second variable position) had over 500, 199, and 86 exact matches, respectively. The unmutated epitope from the A/Puerto Rico/8/34 strain (construct 122) had 4 exact matches in the database, including the original strain, while construct 137 (mutated at the first and the second variable positions) had two exact matches. On the other hand, three mutated epitopes were not naturally occurring: the T-cell epitopes of constructs 132, 135 and 136 (mutated at the third variable position, mutated at the first variable position, and mutated at both the first and third variable position, respectively) had no exact matches in the database.

**Table 1:** Three variable positions were found in the T-helper epitope of post-1934 H1N1 genomes (4th, 6th and 8th residue)

ID	T-helper epitope	Relevance
**122**	WLTEKEGSYP	4 exact matches, including "original" A/Puerto Rico/8/34 epitope

**132**	WLTEKEG**L**YP	No exact matches

**133**	WLTEK**N**GSYP	86 exact matches

**134**	WLTEK**N**G**L**YP	199 exact matches

**135**	WLT**G**KEGSYP	No exact matches

**136**	WLT**G**KEG**L**YP	No exact matches

**137**	WLT**G**K**N**GSYP	2 exact matches

**138**	WLT**G**K**N**G**L**YP	> 500 exact matches, including A/New Caledonia/20/99

All possible mutations were designed and synthesized, and these mutations are bolded for emphasis. The relevance of the constructs was evaluated by using the NCBI protein BLAST analysis to determine if the mutations were naturally occurring, and if so how many influenza genomes encompassed each combination.

To determine if the constructs used shared any similarity with human proteins, the peptides were also screened against the human sequences available in the NCBI BLAST program. There was no significant similarity found for the peptides, thereby eliminating the possibility of any autoimmune reactions should our immunogens be administered to human subjects.

All of the influenza peptides were synthesized in-house by the solid phase method using an automated peptide synthesizer, and high-performance liquid chromatography (HPLC) was used to analyze the sixteen lyophylates. Analysis of the HPLC peaks showed that the purity of the peptide preparations ranged between 90-95% (data not shown).

### MAP constructs more effectively bind antibodies in an ELISA

In order to determine the antibody binding and recognition patterns of our various influenza peptide construct, the individual peptides were tested against commercial influenza strain-specific sheep serum and immune plasma from vaccinated human donors. ELISA plates were coated with the sixteen different influenza peptides, an unrelated HIV gag peptide as negative control, and recombinant hemagglutinin protein from New Caledonia influenza strain. Each condition was coated in triplicate; serum and plasma samples were tested in duplicate under each coating condition, while the third well was left as a blank and subtracted from the average OD value.

**Figure 3:**
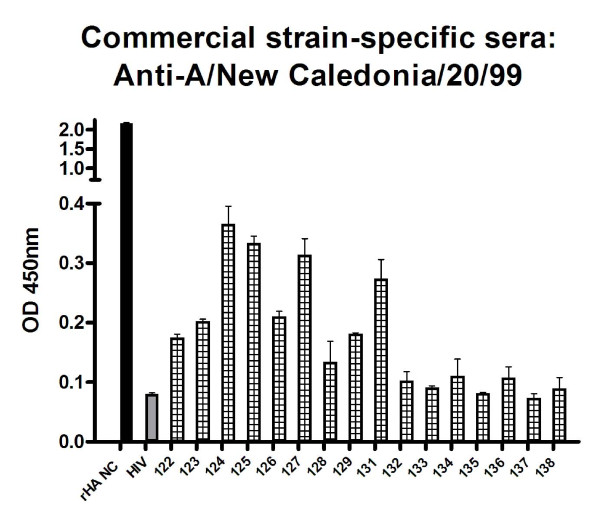
**Ability of sheep serum positive for antibodies against A/New Caledonia/20/99 to bind 16 different influenza T-B and B-T peptides, HIV gag peptide (HIV) and recombinant A/New Caledonia/20/99 hemagglutinin protein (rHA NC) as measured by ELISA**. Absorbance values (OD 450 nm) are given as a mean of duplicates at a 1/100 dilution of sera ± SEM with the subtraction of non-specific binding levels (absorbance value of serum alone control wells). ELISA was run on three separate days and data shown is representative of all results.

New Caledonia strain-specific sheep serum (which is an H1N1 subtype, as the A/Puerto Rico/8/34 strain the peptides are based on) exhibited preferential binding of four peptides: 124, 125, 127 and 131, which are lipidated 2-branched MAP T-B peptide; 4-branched MAP T-B peptide; 4-branched MAP B-T peptide; and lipidated T-B peptide, respectively (Figure 3).

**Figure 4:**
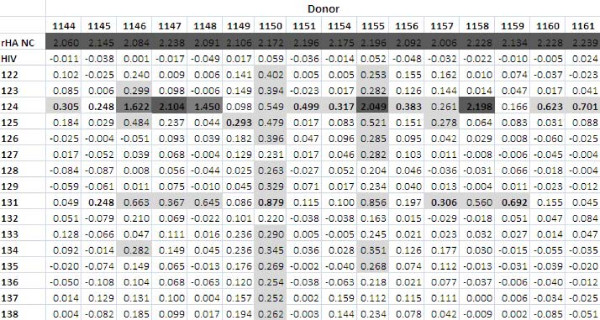
**Summary of ELISA results using plasma from human donors; average OD values of the sixteen donors against the sixteen synthetic influenza peptides and the included controls**. The peptide that resulted in the highest OD value is bolded for each donor, and higher OD values are highlighted (light grey if between 0.250 and 0.99, dark grey if between 1.00 and 1.99 and darkest grey if above 2.00).

**Figure 5:**
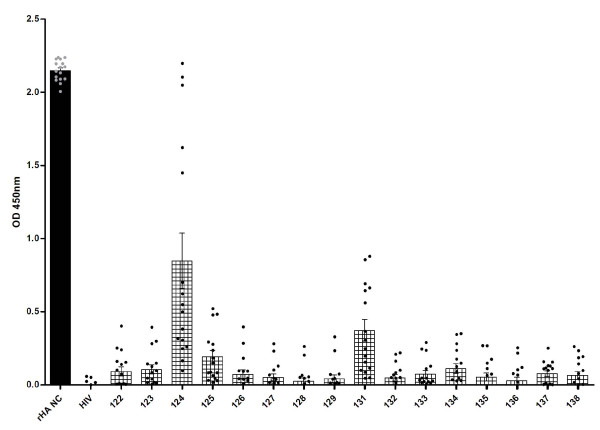
**Global comparison of antibody binding levels of human plasma from 16 different donors against 16 different influenza T-B and B-T peptides, HIV gag peptide (HIV) and recombinant A/New Caledonia/20/99 hemagglutinin protein (rHA NC) as measured by ELISA**. Absorbance values (OD 450 nm) are given as a mean of duplicates at a 1/100 dilution of plasma with the subtraction of non-specific binding levels (absorbance value of plasma alone control wells) ± SEM. Individual absorbance values (as presented in Figure 4) are shown by black circles, while mean OD values (average of all donors) are represented by a bar. The assay was run on three separate days and data shown is representative of all results. All peptides except 128, 129, 132, 135, and 136 had a p-value lower than 0.05 in a Mann Whitney test.

The peptides were screened using immune human samples, and peptide 124 (lipidated 2-branched MAP T-B peptide) showed the most reactivity in terms of antibody binding activity (Figures 4 and 5). Three donors bound 124 (lipidated 2-branched MAP T-B peptide) at exceptionally high rates: the plasma of donors 1147, 1155 and 1158 bound the peptide very strongly (at an OD value above 2.00, which is on par with the OD values of the positive control, the recombinant hemagglutinin protein). An additional two donors, 1146 and 1148, were able to bind peptide 124 at strong rates (OD values between 1.00 and 1.99). Of the remaining donors, all except for four donors bound peptide 124 at a higher rate than all other peptides. Of these, donor 1149 bound peptide 125 (4-branched MAP T-B peptide) at the highest rate, while donors 1150, 1157 and 1159 showed the highest OD value with peptide 131 (lipidated T-B peptide) (Figure 4a). Interestingly, donor 1145 bound both peptides 124 and 131 at the highest rate. Examining mean antibody binding levels, peptide 124 and peptide 131 were clearly preferentially bound even when averaged across all 16 donors (Figure 5). Overall, peptides 124 and 131 (lipidated 2-branched MAP T-B peptide and lipidated T-B peptide, respectively) showed the highest levels of antibody binding.

### Peptides show some ability to bind neutralizing antibodies

To determine if the synthetic influenza peptides were able to bind neutralizing antibodies, we developed two modified functional assays - a competitive microneutralization assay and a competitive plaque reduction assay. Both assays assessed the peptides' ability to inhibit virus neutralization by binding the neutralizing antibodies found in immune samples.

In the competitive microneutralization assay, individual peptides were incubated with human plasma samples, and after one hour of incubation influenza virus was added to the samples. Following an additional incubation time of 1.5 hours, this mix was added to freshly trypsinized MDCK cells, In theory, any virus that had not been neutralized by the immune samples would be free to infect the cells, as evidenced by the detection of newly-formed virus. As such, cells were incubated overnight, and then fixed and lysed. Virus growth was measured by using an antibody directed at the influenza virus nucleoprotein (NP). Note that for each sample a peptide-free control was included, and this represented the baseline level of virus neutralization, which was different for each donor. This baseline level was subtracted from each raw value to normalize results across donors. Lower levels of virus neutralization would result in higher levels of virus growth (as shown by higher OD values), and it was thus possible to determine which peptides were able to bind neutralizing antibodies.

**Figure 6:**
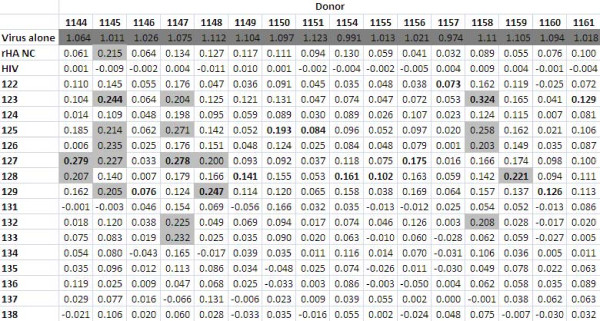
**Average OD values of the sixteen donors against the sixteen synthetic influenza peptides and the included controls as measured by competitive microneutralization assay.** Note that values reflect total background subtraction; adjusted for cells alone control and peptide-free control wells, except the virus alone control which is shown with only cells alone control wells subtracted. The peptide that resulted in the largest change in absorbance value as compared to the peptide-free control is bolded for each donor, and higher differences are highlighted (light grey if between 0.200 and 0.349 and dark grey if above 0.350).

**Figure 7:**
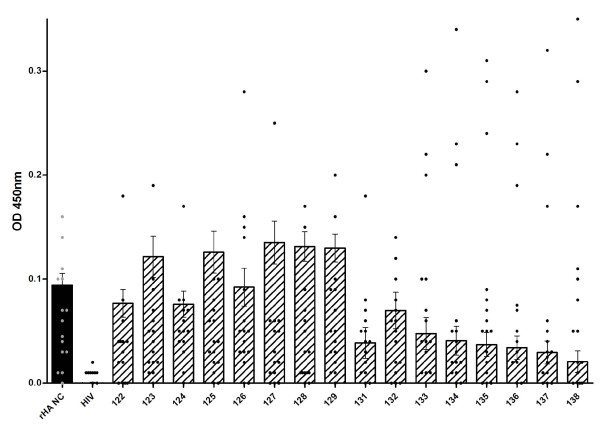
**Global comparison of neutralizing antibody binding levels of human plasma from 16 different donors against 16 different influenza T-B and B-T peptides, HIV gag peptide (HIV) and recombinant A/New Caledonia/20/99 protein (rHA NC), as measured by the inhibition of neutralization of A/Puerto Rico/8/34 influenza virus in a competitive microneutralization assay**. Absorbance values are given as mean of duplicates at a 1/80 dilution of plasma ± SEM. Total background (absorbance of cells alone control and peptide-free control wells) was subtracted from each raw absorbance value. Individual OD values (as presented in Figure 6) are shown by black circles, while mean OD values across all donors (average of all donors) are represented by a bar. All peptides except 131 and 138 had a p-value lower than 0.05 in a Mann Whitney test.

The synthetic influenza peptides were screened by competitive microneutralization assay using immune human plasma, and donors 1145. 1147 and 1158 showed increased reactivity (Figure 6). Examining the average neutralizing antibody binding levels of all 16 donors to each peptide, 5 different designs showed increased immunoreactivity. Peptides 123, 125, 127, 128, and 129 (2-branched MAP T-B peptide, 4-branched MAP T-B peptide, 4-branched MAP B-T peptide, lipidated 2-branched MAP B-T peptide, and T-B peptide with an N-terminal cysteine residue, respectively) were able to bind neutralizing antibodies at a higher rate, as demonstrated by a decrease in virus neutralization (Figure 7).

For the competitive plaque reduction assay, peptides were incubated with influenza strain-specific sheep serum or human plasma samples for one hour. Influenza virus was added to the samples, and after an additional hour of incubation the mixture was added to MDCK cells pre-seeded onto 6-well plates. As in the competitive microneutralization, any virus that had not been neutralized would infect the cells. Infected cells would be lysed, and therefore virus growth could be quantified by counting how many plaques were formed. Cells were thus incubated for 3 days, after which plates were fixed and stained to determine plaque formation under each condition. As before, a no peptide control was included for each sample to determine the baseline level of virus neutralization for each donor and this value was subtracted from each plaque count. Like in the competitive microneutralization assay, higher plaque counts indicated stronger influences of specific peptides on virus neutralization and in turn a more effective binding of neutralizing antibodies.

**Figure 8:**
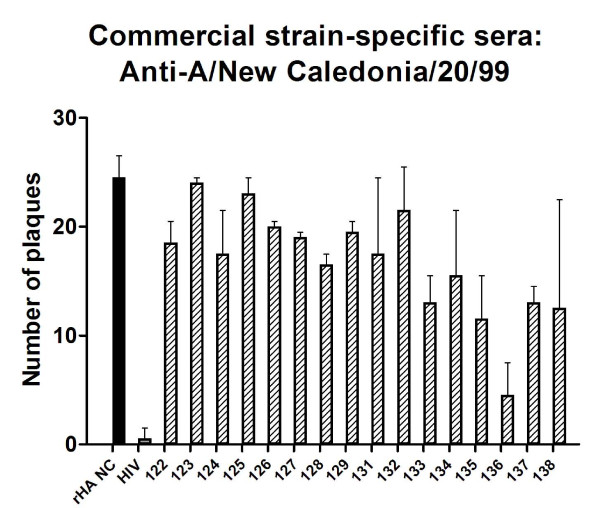
**Ability of 16 different T-B and B-T influenza peptides, HIV gag peptide (HIV) and recombinant A/New Caledonia/20/99 hemagglutinin protein (rHA NC) to bind neutralizing antibodies in sheep serum positive against A/New Caledonia/20/99 strain, and thereby inhibit neutralization of A/New Caledonia/20/99 influenza virus**. Number of viral plaques given as mean of duplicates at a 1/80 dilution of plasma ± SEM. Background (number of plaques in peptide-free serum wells) was subtracted from each plaque count.

All constructs (although peptide 136, which is mutated T-B peptide 5, only to a low extent) were able to inhibit virus neutralization by commercial A/New Caledonia/20/99-strain specific sera (Figure 8). In particular, peptides 123 and 125 (2-branched MAP T-B peptide and 4-branched MAP T-B peptide, respectively) showed slightly higher binding of neutralizing antibodies as compared to other constructs.

**Figure 9:**
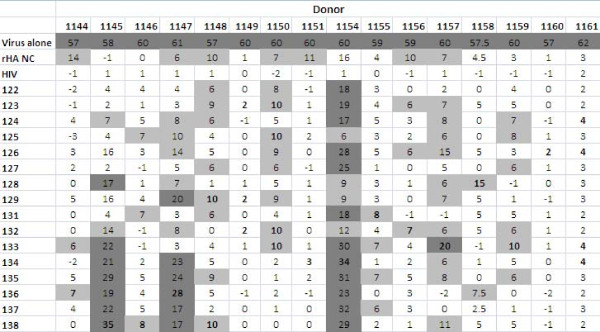
**Average plaque count (after background subtraction, except the virus alone control which is shown directly as counted) of the sixteen donors against the sixteen synthetic influenza peptides and the included controls**. The peptide that resulted in the largest change in plaque count as compared to the peptide-free control is bolded for each donor, and higher differences are highlighted (light grey if between 6 and 15 and dark grey if above 16).

**Figure 10:**
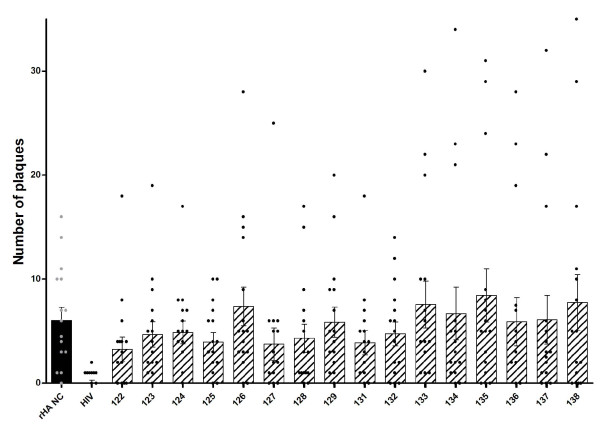
**Global comparison of neutralizing antibody binding levels of human plasma from 16 different donors against 16 different influenza T-B and B-T peptides, HIV gag peptide (HIV) and recombinant A/New Caledonia/20/99 protein (rHA NC), as measured by the inhibition of neutralization of A/New Caledonia/20/99 influenza virus**. Number of viral plaques are given as mean of duplicates at a 1/80 dilution of plasma ± SEM. Background (number of plaques in peptide-free serum wells) was subtracted from each plaque count. Individual plaque counts (as presented in Figure 9) are shown by black circles, while mean plaque counts across all donors (average of all donors) are represented by a bar. All peptides had a p-value lower than 0.05 in a Mann Whitney test.

The assay was conducted with immune human plasma, and donors 1145, 1147 and 1154 were associated with higher general inhibition of virus neutralization upon addition of peptides. It should be noted that the first two of these donors also showed stronger responses in the competitive microneutralization assay. On the other hand, addition of influenza peptides to the plasma of donors 1149, 1151, 1160 and 1161 resulted in very weak changes in levels of virus neutralization (Figure 9). Comparing differences in immunoreactivity between peptides, preferential binding was not limited to one or two designs; peptides 126, 133, 135, and 138 (2-branched MAP B-T peptide, mutated T-B peptide 2, mutated T-B peptide 4, and mutated T-B peptide 6, respectively) all showed increased activity when neutralizing antibody binding was averaged across all donors (Figure 10).

The results of the neutralizing antibody binding assays were not as straightforward as those of the ELISA screening. However, overall peptides 123 and 125 (2-branched MAP T-B peptide and 4-branched MAP T-B peptide, respectively) showed higher ability to bind neutralizing antibodies.

### MAP constructs are able to induce cellular immune responses

The degree to which our synthetic influenza peptides activate cellular immune responses was determined with both cellular proliferation and IFN-γ ELISpot assays.

First, human PBMCs isolated from donor samples were stimulated with individual influenza peptides, an HIV gag peptide (as a negative control), recombinant New Caledonia-strain hemagglutinin protein (rHA), and PMA/ionomycin (as a positive control). Levels of cell division in response to the different stimulants were assessed by thymidine incorporation; dividing cells become radiolabelled and could thus be quantified with the use of scintillation fluid and a luminescence counter. The stimulation index (SI) was then calculated by dividing the average number of cells in each stimulated condition by the average number of cells in unstimulated control wells.

**Figure 11:**
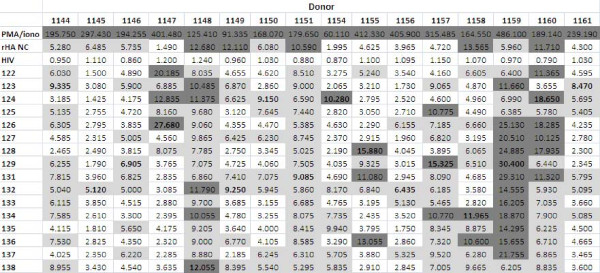
**Average Stimulation Index (ratio of cellular proliferation between stimulated and unstimulated cells) of the sixteen human donors against the sixteen synthetic influenza peptides and the included controls as determined by proliferation assay**. The peptide that resulted in the highest SI value is bolded for each donor, and higher SI values are highlighted (light grey if between 5.000 and 9.999 and dark grey if above 10.000).

Among the sixteen human samples tested, several donors had intermediate SIs across all peptides that were assessed; PBMCs from donors 1145, 1156 and 1161 were not stimulated above an index of 5 by most of the peptides. However, these values were still significantly higher than those associated with the HIV gag peptide-stimulated PBMCs. On the other hand, donors 1148, 1151, 1159 and 1160 showed a five-fold or greater increase in proliferation in response to most peptides. Donor 1159 in particular was a very high responder, with most peptides inducing SI values above 10 (Figure 11).

**Figure 12:**
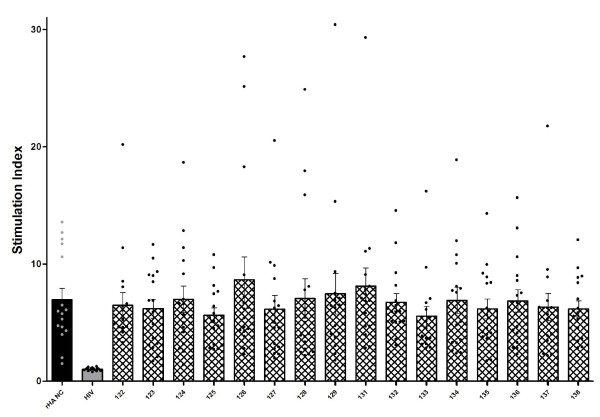
**Global comparison of ability of 16 different influenza T-B and B-T peptides, HIV gag peptide (HIV) and recombinant A/New Caledonia/20/99 hemagglutinin protein (rHA NC) to induce proliferation of human PBMCs, as tracked by [**^**3**^**H] thymidine incorporation**. Stimulants were tested in triplicate and the most outlying value was discarded for each count. Stimulation Index is given as the ratio between the number of cells dividing in response to antigen-stimulation and unstimulated cells ± SEM. Individual SI values (as presented in Figure 8a) are shown by black circles, while mean SI values across all donors is represented by a bar. All peptides had a p-value lower than 0.0001 in a Mann Whitney test.

In terms of peptide-specific differences, there was not a clear bias to a particular peptide. As summarized in Figure 11, lipidated MAP construct 124 (lipidated 2-branched MAP T-B peptide) was able to induce greater than 10-fold increases in levels of proliferation in four donors (donor 1147, 1148, 1154 and 1160 with SI values of 12.835, 11.375, 10.280 and 18.650, respectively), and increases between 5-fold and 9.999-fold in another five donors (donor 1149, 1150, 1151, 1159, and 1159 with SI values of 6.625, 9.150, 6.590, 6.990, and 5.695, respectively). Although peptides 126 and 131 (2-branched MAP B-T peptide and lipidated T-B peptide, respectively) stimulated somewhat higher levels of cellular proliferation (mean SI values across all donors), these were only slight increases compared to the other peptides (Figure 12).

Furthermore, the frequency of IFN-γ secretion in the human PBMCs in response to peptide stimulation was evaluated. As in the proliferation assay, responses to the sixteen individual influenza peptides, an HIV gag peptide (as a negative control), recombinant New Caledonia-strain hemagglutinin protein (rHA), and PMA/ionomycin (as a positive control) were evaluated. Use of a human IFN-γ ELISpot assay allowed quantification of cell-mediated immune responses. Results are presented as average spot forming cell counts (SFC), where average values had been normalized to account for one million cells and spontaneous IFN-γ production (background levels in unstimulated control cells had been subtracted).

**Figure 13:**
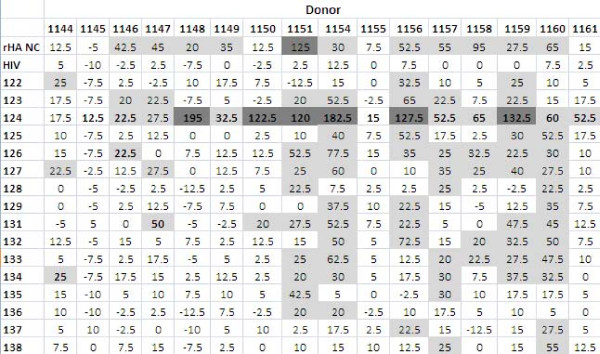
**Average spot-forming cell counts (as determined by ELISpot assay specific for human IFN-γ, per million cells with baseline IFN-γ production in unstimulated cells subtracted) of the sixteen donors against the sixteen different synthetic influenza peptides and the included controls**. The peptide that resulted in the highest SFC value is bolded for each donor, and higher SFC values are highlighted (light grey if between 20 and 99 and dark grey if above 100).

**Figure 14:**
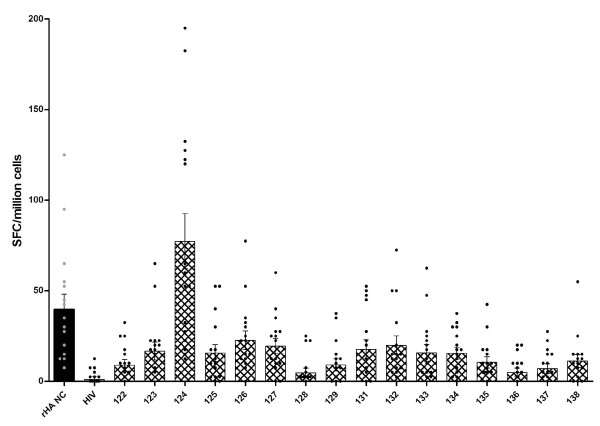
**Global comparison of ability of 16 different influenza T-B and B-T peptides, HIV gag peptide (HIV) and recombinant A/New Caledonia/20/99 (rHA NC) to induce IFN-γ production in human PBMCs**. Stimulants were tested in duplicate, and adjusted average numbers of spot-forming cells (SFC) per one million cells are shown ± SEM (baseline level of IFN-γ production in unstimulated cells was assessed in each donor and subtracted from individual counts). Individual SFC values (as presented in Figure 13) are shown by black circles, while mean SFC values across all donors (average of all donors) are represented by a bar. All peptides except 128, 129, 136, and 137 had a p-value lower than 0.05 in a Mann Whitney test.

Like in the lymphocyte proliferation assay, donors 1145 and 1161 were low responders, with most peptides resulting in SFC counts of less than 20 cells per million (although donor 1156 who had low cellular proliferative responses showed moderate cytokine production). In applying the above criteria that SFC counts of less than 20 cells per million designate low general responders, PBMCs from donors 1144, 1146, 1148, 1149, 1150 and 1155 can also be classified as such, since these donors only produced low levels of IFN-γ in response to most peptides (Figure 13). In stark contrast, donors 1151, 1154, 1156, 1157, 1159 and 1160 were high general responders; stimulation with most peptides resulted in SFC counts higher than 20. It should be noted that donors 1151, 1159 and 1160 also had high general stimulation indexes in the proliferation assay. Peptide 124 (lipidated 2-branched MAP T-B peptide) was able to induce very high levels of IFN-γ production (more than 100 SFC per million cells) among six donors: donors 1148, 1150, 1151, 1154, 1156 and 1159 had counts of 195, 122.5, 120, 182.5, 127.5 and 132.5 SFC per million cells, respectively. It was interesting that peptide 124 elicited high responses even in donors 1148 and 1150, who in general were low responders. Furthermore, in all but two donors stimulation with peptide 124 resulted in IFN-γ production higher than with any other peptide. Only donors 1144 and 1147 did not have the highest peptide-specific response associated with peptide 124. Rather, these two donors showed the best response to peptide 134 (mutated T-B peptide 3) and 131 (lipidated T-B peptide), respectively. Examining peptide-specific cell-mediated responses, it is clear that peptide 124 is the best immunogen as it elicited the highest SFC count when averaged across all the donors (Figure 14). Overall, peptide 124 (lipidated 2-branched MAP T-B peptide) proved to be the best immunogen in both the proliferation and ELISpot assays.

## Discussion

In the field of vaccine development, novel alternatives to traditional immunization approaches are presently being explored; in the case of influenza virus currently available vaccines have several inherent drawbacks, as outlined above. Utilizing short synthetic peptides presents an exciting possibility to replace whole protein or inactivated virus vaccines. Such constructs are attractive options, as synthetic vaccine components can be mass-produced and purified with relative ease, and are fully customizable [29,30]. However, without the addition of powerful adjuvants, synthetic epitope-based vaccines are not highly immunogenic [8,31]. This presents a concern, as only alum and monophosphoryl lipid A (MPL) have been tested for human use, and are still under evaluation [5]. We therefore thought it worthwhile to examine whether the immunogenicity of these potential vaccine alternatives could be improved.

In the present study, we have investigated the immunogenic potential of totally synthetic influenza peptide constructs, consisting of a T-helper epitope and a B-cell epitope of the HA of the A/Puerto Rico/8/34 H1N1 influenza virus strain. It has previously been shown that this linked T-B peptide construct successfully elicits both humoral and cellular immune responses when administered to mice. Although the responses induced by the linked peptide were only moderate, no significant responses were detected in mice immunized with equimolar amounts of non-linked T and B peptides, suggesting that such a conjugated epitope design is a fair starting design to develop synthetic peptides of improved immunogenicity [7]. In an effort to improve the effectiveness of this simple T-B peptide, we used various bioinformatic tools (including sequence databases, software to align and compare sequences, and BLAST analysis) [31] to design and analyze a set of influenza peptides based on the linked epitope backbone. In the studies described herein, several factors that could improve the efficacy of short synthetic peptide immunogens were investigated. Namely, we evaluated lipidation, construction of MAPs, inclusion of a cysteine residue and various mutations for differences in immunoreactivity in both humoral and cellular assays.

In line with previous findings that T epitopes are less variable than B-cell epitopes [32], the sequence used for our T-helper epitope was found to be conserved in all H1N1 strains recorded since 1934. Such non-variable regions are postulated to evade mutations during replication because they have vital viral functions [33], and therefore inclusion of conserved epitopes in a synthetic vaccine may result in broad-spectrum protection against multiple strains and potentially even future variants [32]. Indeed, various studies, especially those focusing on the hypervariable HIV-1 virus, have suggested an advantage in using conserved sequences when designing prophylactic agents [33].

On the other hand, upon aligning post-1934 H1N1 genomes against the B-cell epitope, this region was found to be variable at three positions. All possible mutants were synthesized and evaluated, but the epitope from the A/Puerto Rico/8/34 strain was chosen as the "parent epitope" used for the MAP and/or lipidated constructs, as this sequence has been previously characterized as being immunodominant [34,35].

The NCBI BLAST software employed in the above analysis was also used to compare the epitopes contained in our constructs against the proteins encoded by the human genome. This is an important step when designing peptides, as similar sequences could result in autoimmune reactions upon vaccination of human patients [5,36]. Upon analysing the sixteen influenza peptides and the individual epitope components no similarity whatsoever to the human genome was found, suggesting that our peptide immunogens would not induce adverse self-immune reactions.

In order to assess the likely efficacy of the different B-cell epitope designs in eliciting humoral responses, we screened the various influenza peptides by ELISA that measured antibody binding using sera from immunized sheep and humans. When the peptides were tested against sera from animals immune to A/New Caledonia/20/99 H1N1 strain, peptides 124 (lipidated 2-branched MAP T-B peptide), 125 (4-branched MAP T-B peptide), 127 (4-branched MAP B-T peptide) and 131 (lipidated T-B peptide) bound the antibodies at high rates. Constructs 124, 127, and 131 incorporate two lipid chains, four T-helper epitopes, or one lipid chain respectively, suggesting that increased molecular complexity enhances binding possibly by orienting the B-cell epitope in a more natural configuration. It was not surprising that peptide 127 was among the most immunoreactive in the ELISA, as this design consists of four B-cell epitopes on a T-helper epitope backbone and therefore there were more epitopes present to bind available antibodies. However, this peptide was not associated with the highest binding, perhaps because the added B-cell epitopes were obscuring one another. Upon screening the peptides with samples from immunized human patients, a similar trend arose; constructs 124 (lipidated 2-branched MAP T-B peptide), 125 (4-branched MAP T-B peptide), and 131 (lipidated T-B peptide) once again bound the antibodies at higher rates (at statistically significant levels, with *p *< 0.0001). As in the assays performed with sheep sera, peptide 124 was the most reactive. Interestingly, the constructs with multiple B-cell epitopes did not perform well in the assays utilizing human plasma.

Neutralizing antibodies, specifically those directed at the HA protein, have been characterized as being of major importance in protection against influenza infection [3,32,37,38]. The ELISAs above measured direct antibody binding, but not all these antibodies may be neutralizing; this assay assesses binding of all virus-specific antibodies while only those that actually inhibit viral functions might offer protection [38]. We therefore modified two developed assays [25,26] that quantify functional binding to incorporate a competitive aspect. Binding of neutralizing antibodies was characterized indirectly via inhibition of virus neutralization, whereby a peptide was deemed an effective immunogen if it was able to preferentially bind neutralizing antibodies resulting in decreased virus neutralization by immune samples.

Pre-incubating immune human samples with any of the synthetic influenza peptides prior to testing by competitive microneutralization assay resulted in decreased virus neutralization as compared to the HIV gag peptide control, indicating that neutralizing antibodies were indeed being bound. Specifically, constructs 123, 125, 127, 128, and 129 were associated with larger changes in virus neutralization (*p *< 0.0001). Peptides 125 (MAP with a B-cell epitope backbone four T-helper epitope arms) and 127 (MAP with a T-helper epitope backbone four B-cell epitope arms) were both associated with increased binding activity in at least one of the two types of ELISA screening. Meanwhile, constructs 123 and 128 (branched peptides with a B-cell epitope backbone and two T-helper epitope arms or a T-helper epitope backbone with two lipidated B-cell epitope arms, respectively) are also MAP constructs, in which the B-cell epitopes were presumably found in an orientation similar to that in the native HA protein, a factor that leads to enhanced recognition and binding by neutralizing antibodies. The other lipidated MAP design (peptide 124), which was the most reactive in the ELISA screenings, was not included in the above group of peptides that showed high reactivity. However, this construct was still able to bind neutralizing antibodies, and performed on par with the unmodified peptide (construct 122). Construct 129, with an N-terminal cysteine residue at the T-helper epitope end, was included in this study to address the concern that oxidation of this residue could cause peptides to crosslink via disulfide bonding, resulting in impaired immunoreactivity. Indeed, a previous study found that an air-oxidized T-cell epitope from hen egg-white lysozyme inefficiently stimulated T-cells [39]. However, this peptide contained two cysteine residues within the same epitope which presumably caused intra-peptide bonds (and incorrect folding). The construct in our study, containing a single cysteine residue, seems to be undergoing inter-peptide disulfide bonds. Oxidation thus actually enhanced the immunoreactivity of peptide 129 in functional humoral assays, possibly because cross-linking resulted in heavier molecules. In terms of immunoreactivity in cellular assays, construct 129 proved as efficient or a better immunogen than its non-cysteinated equivalent peptide 122 (refer to Figure 12 and 14). Also of note, all mutant peptides (132 - 138) were associated with decreased virus neutralization relative to the original construct (122). The non-mutated B-cell epitope has previously been characterized as being immunodominant, and therefore these results suggest that the mutations that were incorporated were sufficient to decrease immunoreactivity.

It should be noted that it was necessary to use two different virus strains for the competitive microneutralization assay and the competitive plaque reduction assay. The latter assay was incubated for 3 days, during which the A/New Caledonia/20/99 strain used grew to high enough viral titers to detect infectivity. On the other hand, the competitive microneutralization assay had previously been optimized for overnight incubation. The influenza virus strain used in the competitive plaque reduction assay was tested, but did not grow to detectable levels in this shorter amount of time (data not shown). Thus another influenza H1N1 virus strain (A/Puerto Rico/8/34) was used. However, the results of the competitive plaque reduction assay were still somewhat in agreement with those of the competitive microneutralization assay. When screening the constructs for neutralizing antibody binding activity by competitive plaque reduction assay using sera from sheep immune to the H1N1 strain A/New Caledonia/20/99, again all constructs were able to inhibit virus neutralization relative to the negative control (HIV gag peptide). Furthermore, peptides 123 and 125 gave better results, as determined by a higher plaque count. These two peptides were also among the high binders in the competitive microneutralization assay. When the various peptides were screened using immune human plasma samples, the results were quite a bit different. Once more, all influenza peptides were able to bind neutralizing antibodies to some degree when compared to the HIV gag peptide control. Peptides 123 and 125 (MAP constructs with two or four T-helper epitope arms on a B-cell epitope trunk, respectively) were, as in the previous functional binding assays, more efficient immunogens than peptide 122, the "original" backbone construct. However, these two peptides were not associated with the highest reactivity. Rather, peptides 126, 133, 135, and 138 showed the largest change in plaque count (statistically significant, with *p *< 0.0001, *p *= 0.0004, *p *= 0.0002, and *p *= 0.0027, respectively). Peptide 126 is a MAP construct with two B-cell epitopes, and should in theory be able to bind more neutralizing antibodies simply because there are more available binding sites. However, this peptide had not been among the better immunogens in the other binding assays, whether functional or not. It was quite interesting that three mutated B-cell epitopes were among the higher binding peptides. Although the B-cell sequences of constructs 133 and 138 had been found to have 86 and over 500 matches in the NCBI database, respectively, peptide 135 did not have any exact matches. In other words, a sequence that does not actually occur under natural circumstances was able to effectively bind neutralizing antibodies. It should be stressed that these results are those of the average plaque counts of all 16 human donors. Individual responses were fairly varied, both in terms of which peptides had the largest effect on plaque count and the degree of this effect.

In order to study cellular responses as stimulated by our constructs, the ability of the various synthetic influenza peptides to induce human PBMCs to mount a proliferative response and to produce IFN-γ was investigated. Examining peptide-induced proliferation across all 16 donors, all synthetic influenza T-B dipeptide constructs were effective stimulants (comparable to the recombinant influenza hemagglutinin protein). Stimulation with peptide 126 or 131 (two B-cell epitope arms on a T-helper epitope trunk or lipidated T-B peptide, respectively) led to slightly higher average stimulation (with *p *< 0.0001), however overall cell-mediated immune responses were not substantially high. These results were not surprising as we were working with cells from a varied human donor group. The T-helper epitope included in this study is known to be recognized by murine MHC class II molecule IE^d ^[40]. Moreover, cell-mediated immune responses depend on proper antigen presentation by HLA molecules which are highly polymorphic [36,41]. However, it is reasonable to expect that the T-helper epitope investigated herein can elicit T-cell epitope responses in humans and in the context of several HLA alleles (perhaps more effectively so in association with specific HLA types than with others, as evidenced by the low general stimulation). It is thus reasonable to examine donor-specific responses, and it becomes important to note that the PBMCs from > 50% of patients proliferated at levels higher than 5-fold relative to unstimulated control cells in response to peptide 124 (lipidated 2-branched MAP T-B peptide).We expected this construct to induce a robust proliferative response, as it has two T-helper epitopes that are flanked by lipid moieties. Thus it has multiple and structurally stable epitopes available for presentation, and may therefore be captured by more APCs thereby initiating a stronger cellular immune response.

Examining the IFN-γ secretion showed that peptide 124 is a suitable immunogen. This construct, even when looking at average IFN-γ production in all 16 donors, induces far higher levels of this cytokine than any of the other constructs (*p *< 0.0001).

In summary, although peptide 125 (MAP with four T-helper epitopes on a B-cell backbone) was best across all antibody binding assays, peptide 124 was best overall - this construct performed well in binding assays and resulted in the highest overall cellular immune responses. Thus it appears that the design of construct 124 (lipidated MAP with two T-helper epitopes on a B-cell backbone) is optimal as evaluated herein. The branched and lipidated nature of this molecule may be positioning the B-cell epitope in an optimal conformation, while there are several T-helper epitopes that are lipid-stabilized, leading to proper presentation to multiple T-cells. It would be worthwhile to evaluate peptides of interest *in vivo*, and also possibly to apply similar modifications to other known epitopes of other hypervariable viruses such as HIV and HCV.

## Competing interests

The authors declare that they have no competing interests.

## Authors' contributions

LS carried out the immunological assays and wrote the manuscript. FD participated in the design of the study. AA participated in the design of the study, involved in the analysis of the data and assisted in writing the manuscript. All authors read and approved the final manuscript.
